# Preoperative albumin-to-fibrinogen ratio predicts chemotherapy resistance and prognosis in patients with advanced epithelial ovarian cancer

**DOI:** 10.1186/s13048-019-0563-8

**Published:** 2019-09-18

**Authors:** Wen Yu, Zhongxue Ye, Xi Fang, Xingzhi Jiang, Yafen Jiang

**Affiliations:** Department of Gynecology, HwaMei Hospital, University Of Chinese Academy Of Sciences, NO 41, Xibei Street, Ningbo City, 315000 Zhejiang Province China

**Keywords:** Epithelial ovarian cancer, Prognosis, chemotherapy resistance, Albumin-to-fibrinogen ratio

## Abstract

**Background:**

Epithelial ovarian cancer (EOC) is the majority ovarian cancer (OC) type with a poor prognosis. This present study aimed to investigate potential prognostic factors including albumin-to-fibrinogen ratio (AFR) for advanced EOC patients with neoadjuvant chemotherapy (NAC) followed by debulking surgery.

**Methods:**

A total of 313 advanced EOC patients with NAC followed by debulking surgery from 2010 to 2017 were enrolled. The predictive value of AFR for the overall survival (OS) was evaluated by receiver operating characteristic (ROC) curve analysis. The univariate and multivariate Cox proportional hazards regression analyses were applied to investigate prognostic factors for advanced EOC patients. The association between preoperative AFR and progression free survival (PFS) or OS was determined via the Kaplan–Meier method using log-rank test.

**Results:**

The ROC curve analysis showed that the cutoff value of preoperative AFR in predicting OS was determined to be 7.78 with an area under the curve (AUC) of 0.773 (*P* < 0.001). Chemotherapy resistance, preoperative CA125 and AFR were independent risk factors for PFS in advanced EOC patients. Furthermore, chemotherapy resistance, residual tumor and AFR were significant risk factors for OS by multivariate Cox analysis. A low preoperative AFR (≤7.78) was significantly associated with a worse PFS and OS via the Kaplan–Meier method by log-rank test (*P* < 0.001).

**Conclusions:**

A low preoperative AFR was an independent risk factor for PFS and OS in advanced EOC patients with NAC followed by debulking surgery.

## Introduction

Epithelial ovarian cancer (EOC), as the majority ovarian cancer (OC) type, represents the most lethal gynecologic malignancy [1] and the 5th leading cause of cancer-related death worldwide [2]. Despite the improved therapeutic strategies, OC is still a deadly disease with a mortality rate over 30% [3]. As reported by previous studies, the median progression-free survival (PFS) is only 16–22 months and the 5-year overall survival (OS) rate is less than 30% [4]. Unfortunately, most EOC cases were diagnosed at advanced stages by reason of its asymptomatic rapid progression. Over the last decades, the primary standard treatment involves cytoreductive surgery and platinum/taxane based chemotherapy [5]. However, the the overall survival of EOC remains poor due to the advanced stage at diagnosis, chemoresistance and high tumor recurrence rate [6]. Therefore, to investigate potential predictors is urgently needed for the improvement of diagnosis, new therapeutic target, disease activity and treatment response monitoring.

Recently, a great deal of evidence has shown that the status of systemic inflammatory response to tumor and nutrition closely correlates with the prognosis in various cancers [7, 8]. Albumin (Alb), which has been commonly used to for the nutritional and inflammatory status assessment, is indicated as a potential prognostic factor for advanced cancer [9]. Increasing evidence has also revealed the close association between coagulation and tumor progression and metastasis [10, 11]. Fibrinogen (Fib), which is an essential protein in the coagulation cascade and an acute-phase reaction protein for systemic inflammation like C-reactive protein (CRP), plays an important role in tumor development [12]. Albumin-to-fibrinogen ratio (AFR), which takes both Alb and Fib into account, has been indicated as a prognostic factor for various malignancies including non-small cell lung cancer (NSCLC) [13], chronic lymphocytic leukemia [14], and breast cancer [15], etc. However, no study has investigated the association between AFR and EOC until now. Hence, the aim of this study was to investigate whether circulating AFR could serve an effective prognostic biomarker for EOC.

## Material and methods

### Patients

This retrospective study was approved by the Medical Institutional Ethics Committee of Hwa Mei Hospital and Zhejiang province. Eligible subjects with newly diagnosed EOC admitted at the Department of Gynecology, Hwa Mei Hospital, University Of Chinese Academy Of Sciences from 2010 to 2017 were enrolled into this study population. The inclusion criteria were as follows: (a) newly histologically diagnosed advanced EOC patients with the International Federation of Gynecology and Obstetrics (FIGO) stage III or IV; (b) available preoperative serum expressions of Alb and Fig; (c) patients who underwent neoadjuvant chemotherapy (NAC) followed by debulking surgery; (c) patients with adequate clinicopathological data and (d) patients with a follow up for at least 12 months. The exclusion criteria were as follows: (a) patients who had clinical evidence of infection or inflammatory conditions; (b) patients who were with other malignancies; (c) patients who were with autoimmune or hematological diseases, abnormal liver or renal function and (d) patients who were lost to follow-up or without complete data. Informed consent was obtained from all the enrolled patients who underwent cytoreduction and chemotherapy.

### Treatment and data collection

A same experienced pathologist who was blinded to this study was invited to review all the surgical specimens and make the pathological evaluation. All the included patients underwent three courses of adjuvant first-line platinum-based chemotherapy before the primary cytoreduction surgery. Each enrolled patient was required to perform the examinations of whole body computed tomography (CT) or positron emission tomography/computed tomography (PET/CT). The tumor stage was determined for all patients following the guidance FIGO system. Demographics, clinicpathological parameters and preoperative laboratory tests were collected from medical records. Preoperative fasting peripheral blood samples were obtained on 1 day prior to operation and were then processed within 48 h for laboratory variable detections. The blood cells, C-reactive protein (CRP), Alb, Fib and CA125 were detected using the obtained blood samples. The AFR was calculated by Alb (g/L)/Fib (g/L) ratio.

### Prognosis definition

At each visit during follow up, clinical, imaging examinations and the serum CA-125 expression were assessed for treatment evaluation. The primary end point of this study was PFS, overall survival (OS), or the last follow up (December, 2017). The chemotherapy responses was assessed following the guidance of the Response Evaluation Criteria in Solid Tumors (RECIST) [16]. Chemotherapy sensitivity was defined as complete response (CR) or partial response (PR), whereas chemotherapy resistance was defined as stable disease (SD) or progressive disease (PD). PFS was defined as the duration from the date of initial surgery to the evidence of tumor progression, death or follow up deadline. OS was defined as the duration from the date of initial surgery to death or the last follow-up.

### Statistical analysis

Statistical analyses were performed with the software of GraphPad prism (version. 5.0, GraphPad Inc., San Diego, CA, USA) and SPSS (version 19.0, SPSS Inc., Chicago, IL, USA). Data are presented with number with proportion, or mean ± standard deviation (SD). The predictive value of AFR for the OS as well as the cut-off value was evaluated by receiver operating characteristic (ROC) curve analysis and the Youden index. The comparisons between categorical variables were performed with the Chi-square test, while continuous variables with Student t test or Mann–Whitney U test as appropriate. The univariate and multivariate analyses were performed to evaluate the prognostic factors using the Cox proportional hazard model and only those factors with a *p* value < 0.1 in the univariate mode were further analyzed in the multivariate mode. OS and PFS were calculated using Kaplan–Meier method and the log-rank test. A two-sided *P* < 0.05 was accepted as statistically different.

## Results

### Patient characteristics

**Fig. 1:**
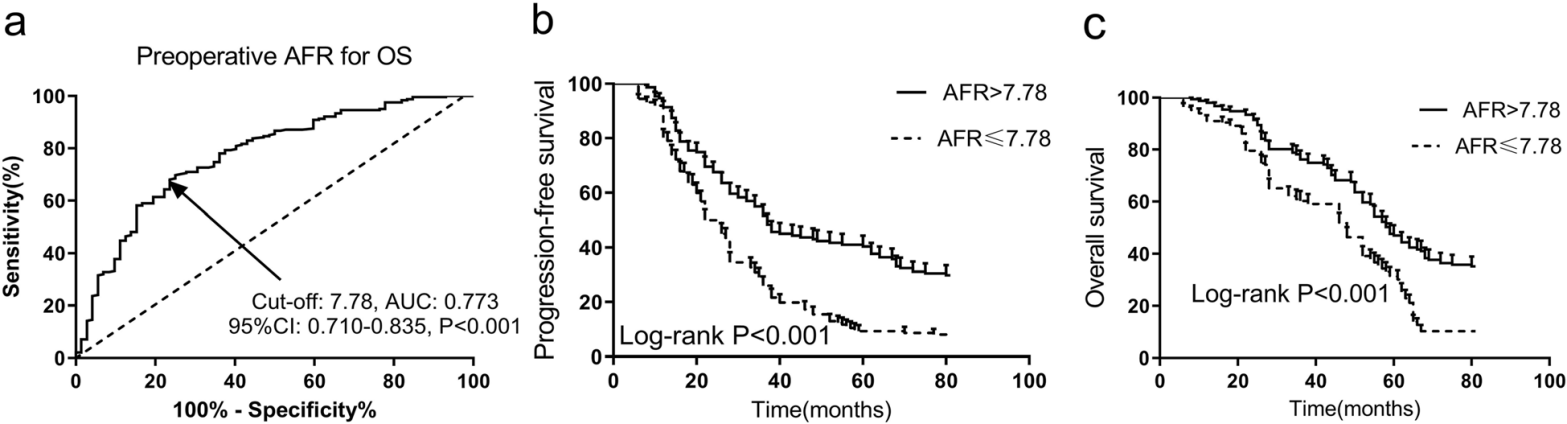
AFR and prognosis in advanced EOC patients. **a** Predictive value of preoperative AFR for OS in advanced EOC by ROC curve. The cutoff value of AFR in predicting OS was determined to be 7.78 with an AUC of 0.773, 95% CI of 0.710–0.835, a sensitivity of 64.32% and a specificity of 77.78%, respectively (*P* < 0.001); (**b**) PFS in advanced EOC patients according to AFR by Kaplan-Meier curve analysis. Patients with a lower preoperative AFR (≤7.78) had a worse PFS than those with a high preoperative AFR (> 7.78) by log-rank test (*P* < 0.001); (**c**) OS in advanced EOC patients according to AFR by Kaplan-Meier curve analysis. Patients with a lower preoperative AFR (≤7.78) had a worse OS than those with a high preoperative AFR (> 7.78) by log-rank test (*P* < 0.001). AFR, albumin-to-fibrinogen ratio; PFS, progression-free survival; OS; overall survival; EOC, epithelial ovarian cancer; ROC, receiver operating characteristic; AUC, the area under the curve; CI, confidence interval

A total of 375 subjects were primarily enrolled according to the inclusion criteria and 62 were excluded due to the exclusion criteria (10 had infection or inflammatory conditions, 12 were with other malignancies, 13 were with autoimmune or hematological diseases, 20 were with abnormal liver or renal function and 27 were lost to follow-up or without complete data). Finally, 313 advanced EOC patients with a mean age of 64.4 years were included the analysis. As shown in Fig. 1a, the ROC curve analysis showed that the cutoff value of AFR in predicting OS was determined to be 7.78 with an area under the curve (AUC) of 0.773, 95% CI of 0.710–0.835, a sensitivity of 64.32% and a specificity of 77.78%, respectively (*P* < 0.001). According to the baseline cutoff value, all the 313 were categorized into two groups, 151 (48.2%) in the low-AFR group (AFR ≤ 7.78) and 162 (51.8%) in the high-AFR group (AFR > 3.45). The baseline demographic and clinicopathological characteristics associated with AFR are detailed exhibited in Table 1. In comparison with the low-AFR group, patients in the high-AFR group showed a lower age (*P* = 0.004), ECOG PS (*P* = 0.016), histological grade (*P* = 0.042) and FIGO stage (*P* = 0.032). High AFR expression was significantly associated with an increased incidence of first-line platinum-based chemotherapy resistance (*P* = 0.033). Patients with preoperative AFR ≤7.78 had a higher prevalence of postoperative residual tumor larger than 1 cm (*P* = 0.005). However, no statistically differences were noted between the high-AFR and low-AFR groups in respect to BMI, smoking and alcohol habits, preoperative comorbidities and histological type (*P* > 0.05). Other laboratory tests including blood cells analyses, CRP and CA125 were also analyzed. As shown in Table 2, the results indicated that high AFR expression was significantly associated with lower expressions of CRP (*P* = 0.033) and CA125 (*P* = 0.002). No significant differences were observed in blood cell analyses between low-AFR and high-AFR groups (*P* > 0.05).

**Table 1:** Clinicpathological parameters associated with AFR in advanced EOC patients

	AFR		
Parameters	Low (≤7.78)	High (> 7.78)	*p* value
Number	151	162	–
Age (years)	65.8 ± 8.7	63.1 ± 7.8	0.004*
BMI (kg/m^2^)	23.1 ± 2.3	23.4 ± 2.1	0.229
Current smokers (n, %)	18 (11.9)	24 (14.8)	0.453
Alcohol (n, %)	14 (9.3)	19 (11.7)	0.479
Comorbidities (n, %)			–
Diabetes	22 (14.6)	17 (10.5)	0.275
Hypertension	27 (17.9)	22 (13.6)	0.295
Hyperlipemia	15 (9.9)	12 (7.4)	0.426
Histological grade (n, %)			0.042*
G1	76 (50.3)	100 (61.7)	–
G2/3	75 (49.7)	62 (38.3)	–
Histological type (n, %)			0.444
Serous	106 (70.2)	120 (74.1)	–
Non-serous	45 (29.8)	42 (25.9)	–
FIGO stage (n, %)			0.032*
III	113 (74.8)	137 (84.6)	–
IV	38 (25.2)	25 (15.4)	–
ECOG PS (n, %)			0.016*
0	101 (66.9)	128 (79.0)	–
≥ 1	50 (33.1)	34 (21.0)	–
Chemotherapy resistance (n, %)	54 (35.8)	40 (24.7)	0.033*
Residual tumor (cm)			0.005*
≤ 1	100 (66.2)	130 (80.2)	–
> 1	51 (33.8)	32 (19.8)	–

*EOC*, Epithelial ovarian cancer; *BMI*, Body mass index; *FIGO*, International Federation of Gynecology and Obstetrics; *AFR*, Albumin-to-fibrinogen ratio; *ECOG PS*, Eastern cooperative oncology group performance status. *P*-values were calculated by Student’s t test, Mann–Whitney U test or Chi-squared test. * *P* < 0.05.

**Table 2:** Laboratory tests associated with AFR in advanced EOC patients

	AFR		
Laboratory tests	Low (≤7.78)	High (> 7.78)	*p* value
Hemoglobin (g/L)	102.4 ± 11.5	104.1 ± 12.5	0.212
Platelet (10^9^/L)	164.4 ± 35.8	170.7 ± 41.3	0.152
WBC(10^9^/L)	7.0 ± 1.9	7.2 ± 2.2	0.392
Albumin (g/L)	38.9 ± 4.6	39.2 ± 3.9	0.533
Total protein (g/L)	70.1 ± 7.8	69.6 ± 6.5	0.537
Total cholesterol (mmol/L)	4.3 ± 0.6	4.2 ± 0.7	0.177
Triglyceride (mmol/L)	1.2 ± 0.5	1.3 ± 0.5	0.078
CRP (ng/L)	7.4 ± 10.1	5.3 ± 7.1	0.033*
CA125 (U/mL)	145.1 ± 189.3	87.2 ± 128.1	0.002*

*WBC*, White blood cell; *CRP*, C-reactive protein; *EOC*, Epithelial ovarian cancer; *AFR*, Albumin-to-fibrinogen ratio. *P*-values were calculated by Student’s t test or Mann–Whitney U test. * *P* < 0.05.

### Risk factors for PFS and OS in advanced EOC patients

As shown in Table 3, potential risk factors for PFS in advanced EOC patients were assessed by univariate and multivariate Cox proportional regression analyses. Potential risk factors with a *P* value < 0.1 by the univariate Cox analysis including Age, histological grade, FIGO stage, chemotherapy resistance, residual tumor, CA125 and AFR were enrolled into the multivariate Cox analysis. The results indicated that chemotherapy resistance (HR: 1.84, 95 CI%: 1.12–2.88, *P* = 0.032), CA125 (HR: 1.42, 95 CI%: 1.02–2.05, *P* = 0.042) and AFR (HR: 1.38, 95 CI%: 1.04–1.82, *P* = 0.019) were independent risk factors for PFS in advanced EOC patients. Furthermore, chemotherapy resistance (HR: 1.30, 95 CI%: 1.01–1.67, *P* = 0.032), residual tumor (HR: 1.48, 95 CI%: 1.01–2.21, *P* = 0.046) and AFR (HR: 2.19, 95 CI%: 1.18–3.67 *P* = 0.021) were significant risk factors for OS in advanced EOC patients by multivariate Cox analysis (Table 4).

**Table 3:** Risk factors for PFS by univariate and multiple Cox regression analysis

	Univariate		Multivariate	
Parameters	HR(95% CI)	*p* value	HR(95% CI)	*p* value
Age (high vs low)	1.50 (1.11–2.08)	0.016*	1.17 (0.85–1.62)	0.301
BMI (high vs low)	0.82 (0.61–1.15)	0.251		
Current smokers (yes vs no)	1.28 (0.97–1.70)	0.112		
Alcohol (yes vs no)	1.16 (0.89–1.53)	0.247		
Diabetes (yes vs no)	1.03 (0.75–1.41)	0.822		
Hypertension (yes vs no)	1.06 (0.73–1.49)	0.794		
Hyperlipemia (yes vs no)	0.85 (0.42–1.65)	0.654		
Histological grade (G2/3 vs G1)	1.76 (1.19–2.63)	0.012*	1.63 (0.92–2.77)	0.093
Histological type (serous vs non-serous)	1.21 (0.89–1.59)	0.179		
FIGO stage (IV vs III)	1.72 (1.09–2.73)	0.024*	1.44 (0.79–2.69)0.254	
ECOG PS (≥1 vs 0)	0.86 (0.71–1.07)	0.852		
Chemotherapy resistance (yes vs no)	2.07 (1.35–3.17)	0.011*	1.84 (1.12–2.88)	0.032*
Residual tumor (> 1 cm vs ≤1 cm)	2.01 (0.94–4.31)	0.071	1.61 (0.91–2.85)	0.121
Hemoglobin (high vs low)	1.84 (0.69–4.78)	0.211		
Platelet (high vs low)	1.55 (0.38–5.78)	0.529		
WBC (high vs low)	1.86 (0.86–3.89)	0.127		
Albumin (high vs low)	0.92 (0.57–1.42)	0.721		
Total protein (high vs low)	0.81 (0.44–1.48)	0.472		
Total cholesterol (high vs low)	0.75 (0.47–1.24)	0.261		
Triglyceride (high vs low)	1.26 (0.70–2.22)	0.425		
CA125 (high vs low)	1.72 (1.22–2.44)	0.011*	1.42 (1.02–2.05)	0.042*
CRP (high vs low)	1.36 (0.69–2.64)	0.373		
AFR (≤7.78 vs > 7.78)	1.56 (1.21–2.05)	0.007*	1.38 (1.04–1.82)	0.019*

*EOC*, Epithelial ovarian cancer; *PFS*, Progression-free survival; *BMI*, Body mass index; *FIGO*, International Federation of Gynecology and Obstetrics; *AFR*, Albumin-to-fibrinogen ratio; *ECOG PS*, Eastern cooperative oncology group performance status; *WBC*, White blood cell; *CRP*, C-reactive protein; *HR*, Hazard ratio; *CI*, Confidence interval. * *P* < 0.05.

**Table 4:** Risk factors for OS by univariate and multiple Cox analysis

	Univariate		Multivariate	
Parameters	HR(95% CI)	*p* value	HR(95% CI)	*p* value
Age (high vs low)	1.23 (0.84–1.81)	0.304		
BMI (high vs low)	0.53 (0.24–1.15)	0.112		
Current smokers (yes vs no)	1.09 (0.76–1.57)	0.647		
Alcohol (yes vs no)	1.17 (0.82–1.67)	0.384		
Diabetes (yes vs no)	1.24 (0.71–2.13)	0.435		
Hypertension (yes vs no)	1.31 (0.83–2.05)	0.247		
Hyperlipemia (yes vs no)	0.85 (0.54–1.33)	0.468		
Histological grade (G2/3 vs G1)	1.57 (1.03–2.41)	0.034*	0.92 (0.81–1.22)	0.068
Histological type (serous vs non-serous)	0.49 (0.83–2.64)	0.181		
FIGO stage (IV vs III)	1.89 (1.17–3.11)	0.014*	1.58 (0.77–3.35)	0.221
ECOG PS (≥1 vs 0)	1.12 (0.74–1.69)	0.603		
Chemotherapy resistance (yes vs no)	1.27 (1.03–1.62)	0.021*	1.30 (1.01–1.67)	0.032*
Residual tumor (> 1 cm vs ≤1 cm)	1.32 (1.04–1.68)	0.022*	1.48 (1.01–2.21)	0.046*
Hemoglobin (high vs low)	0.78 (0.39–1.56)	0.478		
Platelet (high vs low)	1.55 (0.75–3.18)	0.237		
WBC (high vs low)	1.39 (0.77–2.51)	0.278		
Albumin (high vs low)	0.65 (0.38–1.12)	0.112		
Total protein (high vs low)	0.89 (0.55–1.45)	0.642		
Total cholesterol (high vs low)	0.69 (0.31–1.52)	0.359		
Triglyceride (high vs low)	1.50 (0.83–2.73)	0.181		
CA125 (high vs low)	1.41 (0.73–2.73)	0.304		
CRP (high vs low)	1.17 (0.64–2.11)	0.613		
AFR (≤7.78 vs > 7.78)	2.50 (1.44–4.09)	0.007*	2.19 (1.18–3.67)	0.021*

*EOC*, Epithelial ovarian cancer; *OS*, Orall survival; *BMI*, Body mass index; *FIGO*, International Federation of Gynecology and Obstetrics; *AFR*, Albumin-to-fibrinogen ratio; *ECOG PS*, Eastern cooperative oncology group performance status; *WBC*, White blood cell; *CRP*, C-reactive protein; *HR*, Hazard ratio; *CI*, Confidence interval. * *P* < 0.05.

### PFS and OS associated with AFR

To further analyze the prognostic values of preoperative AFR in advanced EOC patients, the Kaplan-Meier curves of PFS and OS are then performed. As shown in Fig. 1b and c, a low preoperative AFR (≤7.78) was significantly associated with a worse PFS and OS by log-rank test (*P* < 0.001).

## Discussion

In this present retrospective study, lower AFR was found to be significantly correlated with elder age, higher histological grade, FIGO stage, ECOG PS, larger residual tumor and higher incidence of chemotherapy resistance after cytoreduction surgery in advanced EOC. Furthermore, low preoperative AFR level and chemotherapy resistance were shown to be two independent risk factors associated with a worse PFS and OS based on the multivariate Cox regression model.

To the best of our knowledge, no paper regarding AFR and prognosis in advanced EOC patients has been published until now. This study is the first to suggest AFR as a prognostic factor in advanced EOC patients who underwent neoadjuvant chemotherapy (NAC) followed by debulking surgery. Previously published data in patients with high grade serous ovarian cancer (HGSOC) has indicated that clinical resistance to first-line chemotherapy is significantly associated with a shorter PFS and OS [17]. Our results were in accordance with their conclusions, reflecting the close correlation between chemotherapy resistance and prognosis. Our results has also indicated preoperative AFR as an independent prognostic factor for advanced EOC patients. Previous studies conducted in non-small cell lung cancer individuals have suggested circulating AFR as an effective biomarker to predict prognosis [18], which is in support of our results. Preoperative FAR was a strong independent prognostic factor in breast cancer.

A great deal of evidence has reported that the nutritional and systemic inflammatory status were closely associated with prognosis in patients with cancers [7, 8]. Furthermore, nutrition and inflammation are closely regulated with each other instead of being independent from each other [19]. As an important indicator of nutrition and inflammatory state, the low expression of serum Alb is reported to be significantly associated with poor prognosis in various malignancies including prostate cancer [20], lung cancer [21], breast cancer [15] and chronic lymphocytic leukemia [22]. The close correlation between inflammation and tumorigenesis may be the main possible explanation for the prognostic role of serum Alb in various malignancies [15]. Elevated serum Fib has also been indicated as a predictor for poor prognosis in esophageal cancer [23], breast cancer [24] and EOC [25]. Increased plasma fib expression contributes to elevated blood coagulability state and promotes the intravasation, adhesion and survival of tumor cells, which results in increased metastatic potential in cancer model [26]. Elevated serum Fib and decreased serum Alb have been widely accepted as useful biomarkers for elevated systemic inflammation [19, 27]. Furthermore, AFR, a ratio of Alb to Fib, could amplify the sensitivity of inflammation and nutrition status in EOC patients and it was superior to predict prognosis of EOC in comparison with single Alb or Fib.

Although this was the first study to explore the prognostic role of AFR in advanced EOC patients, it had several limitations. First, this was a retrospective, single-center study with relatively small sample size. Second, our conclusions were not validated yet and further study is needed. Third, the AFR expressions were only assessed before the operation, whether postoperative AFR could also serve as a prognostic factor remains unclear. The last, the involved mechanisms for the prognostic role of preoperative AFR for advanced EOC patients were not verified.

## Conclusions

In conclusion, our results indicated that preoperative AFR could serve as a potential predictor for the prognosis in advanced EOC patients with NAC followed by debulking surgery. However, a prospective multiple-central cohort with a larger sample size are warranted to validate the clinical application of AFR in advanced EOC.

## Data Availability

Please contact the corresponding author Yafen Jiang (jiangyafennb@sina.com).

## Abbreviations

AFR: Albumin-to-fibrinogen ratio
AUC: Area under the curve
BMI: Body mass index
CI: Confidence interval
CRP: C-reactive protein
ECOG PS: Eastern cooperative oncology group performance status
EOC: Epithelial ovarian cancer
FIGO: International Federation of Gynecology and Obstetrics
HGSOC: High grade serous ovarian cancer
HR: Hazard ratio
NAC: Neoadjuvant chemotherapy
OC: Ovarian cancer
OS: Overall survival
PFS: Progression-free survival
ROC: Receiver operating characteristic
WBC: White blood cell
